# Bullying Victimization and Mattering: An Analysis of Adolescent Gender Identity and Sexual Orientation

**DOI:** 10.3390/children13081066

**Published:** 2026-08-11

**Authors:** Sarah Hobson, Amanda Krygsman, Heather Brittain, Anjalika Khanna Roy, Tracy Vaillancourt

**Affiliations:** 1 School of Psychology, Faculty of Social Science, University of Ottawa, 120 University Private, Ottawa, ON K1N 6N5, Canada; 2 Counselling Psychology, Faculty of Education, University of Ottawa, 145 Jean-Jacques-Lussier Private, Ottawa, ON K1N 6N5, Canada

**Keywords:** mattering, bullying victimization, intersectionality, gender identity, sexual orientation, adolescents

## Abstract

**Highlights:**

**What are the main findings?**

• Greater bullying victimization was associated with lower perceptions of mattering to others across most intersecting gender identity and sexual orientation groups, with the strongest negative association observed among straight girls.
• Sexually and/or gender diverse youth generally reported lower average perceptions of mattering than more socially advantaged groups (i.e., straight girls, straight boys), outside of bullying experiences.

**What are the implications of the main findings?**

• Lower perceptions of mattering among bullied and gender and/or sexually marginalized youth may be associated with poorer academic, physical, and psychological outcomes.
• Societal and social efforts that communicate youth’s importance and value to others may help mitigate these harmful effects, especially for bullied and gender and/or sexually marginalized youth.

**Abstract:**

**Background/Objectives:** Youth who experience bullying victimization often report lower mattering, the perception of being valued by others; yet this association remains underexplored across intersections of gender and sexual identities. **Methods:** Using Spring 2025 data from the Health and Peer Relations Study, we examined whether gender identity and sexual orientation moderated the association between bullying and mattering among Canadian youth in Grades 7–12 (*N* = 54,181; racially/ethnically diverse = 65.2%, sexually diverse = 14.8%, gender diverse = 3.5%), controlling for grade and race/ethnicity. **Results:** After controlling for covariates, the three-way interaction between bullying, gender identity, and sexual orientation was statistically significant (*p* = 0.042). Bullying was negatively associated with mattering across most groups, and the association was strongest among straight girls, while associations were largely similar across other groups. In contrast, average mattering followed a clear social gradient wherein straight girls and boys reported the highest levels, and sexually and/or gender diverse youth reported the lowest. **Conclusions:** Bullying is associated with lower mattering among most youth. However, socially advantaged youth reported stronger associations between bullying and mattering, while socially marginalized youth reported lower mattering regardless of bullying exposure. Findings highlight the importance of addressing youth bullying and underlying drivers of social inequities in mattering.

## 1. Introduction

Adolescence is a period in which young people come to understand who they are in relation to others, including how their identities are seen and valued [1]. For youth with diverse gender identities and sexual orientations, this journey can involve experiences of exploration, belonging, as well as marginalization and bullying victimization [2,3]. These experiences are not simply a function of individual identities, but reflect broader societal values, norms, practices, and systems of power that shape how certain identities are perceived and treated. Within these systems, intersecting identities position youth differently in social hierarchies, influencing how they are responded to by their peers and how they relate to themselves. These dynamics are especially salient in school contexts, where persistent disparities in the experiences of gender and sexually diverse youth have been well documented [4,5,6,7,8]. Addressing these inequities is not only critical for promoting positive developmental outcomes but also reflects a broader ethical imperative to ensure safe and equitable educational environments for all youth. As such, research that centers on gender identity and sexual orientation is critical for understanding and reducing these disparities. Accordingly, we examined how bullying victimization experiences and intersecting gender identity and sexual orientation were associated with perceptions of mattering to others.

Mattering refers to an individual’s tendency to perceive themselves as of value to others, particularly to specific people in their social lives (e.g., peers, friends, parents) [9,10], including perceptions that others actively pay attention to them, regard them as important, depend on them, and care about them [11,12]. Perceptions of mattering are formulated through social interactions and relationships with others [9,10,12]. Although conceptualized as a universal need [10], concerns with mattering to others are thought to be especially salient during adolescence [10,12]. Compared with childhood, adolescence is a period of active identity formation and development [13,14]. During this developmental period, adolescents place an increased emphasis on social information and self-relevant feedback [15,16] that informs their developing sense of identity [1]. As adolescents become increasingly concerned with how they are perceived and valued by others, experiences of mattering may represent a particularly informative source of self-information [17]. Consequently, mattering is considered a core component of self-concept [9,10,11] and reflects adolescents’ perceptions of their own value and significance to others, making it highly relevant to identity development, self-worth, and social belonging [17]. Given the developmental salience of identity formation and social feedback during adolescence, mattering has been proposed as an important developmental asset and is consistently associated with positive psychosocial adjustment, including greater self-esteem [18], wellness [19], and well-being [17,20].

### 1.1. Mattering and Bullying Victimization

Given the developmental importance of mattering during adolescence, interactions and relationships with peers may be particularly relevant to adolescents’ perceptions of whether they are valued and significant to others. Youth become increasingly oriented toward peer relationships with age [21,22], especially during adolescence [15,23]. Adolescents place considerable importance on peer appraisal and other forms of self-relevant social feedback [15,24,25,26] as they seek greater autonomy and learn about themselves in non-familial contexts [13,26,27]. Accordingly, peers assume a central role in socioemotional development outside of the home [21,28,29]. Consequently, the tone and valence of peer interactions may represent an important source of information for adolescents’ core self-evaluations and self-concept [30,31], including their perceptions of value to others.

Negative interpersonal experiences, such as bullying victimization, may be particularly relevant to adolescents’ mattering beliefs [32,33,34]. Bullying is strategic, repeated, aggressive behaviour perpetrated by one (or more individuals) who wields more power than their target [35]. Youth can be bullied through various means, including physical, verbal, relational, and electronic acts of aggression [36,37]. Bullying experiences are socially painful [38] and psychologically [39] and physiologically stressful [40]. Consistent evidence shows that bullying undermines self-esteem and self-conceptualization [41,42], especially for those more frequently targeted [42]. Given that mattering is a key component of the self-concept that is formulated through social exchanges [9,10,12,43,44], ongoing, hostile peer interactions can have serious implications for youth’s sense of mattering [33,45].Globally, bullying is a common problem and significant threat to young people, with nearly one-third of youth experiencing some form of bullying victimization at least once a month [46]. In Canada, where the study was conducted, Canada placed 23rd of 38 economically advanced countries on bullying victimization, with one in five Canadian youth (20%) reporting that they had been bullied [47].

Few studies have examined the relation between youth bullying exposure and sense of mattering [32,34], with preliminary findings suggesting a negative impact of such experiences on youth perceptions of mattering. In a subsample of 400 American Indian and Alaska Native youth, Edwards et al. [32] found that those who were bullied at school or online reported lower feelings of mattering to their school. Siller et al. [34] extended these findings using latent class analyses to examine distinct classes of interpersonal violence victimization/perpetration on the sense of mattering of 2049 middle and high school youth. Siller et al. [34] found that youth with lower school mattering were more likely to have been bullied compared to those with minimal to no experience of interpersonal victimization/perpetration.

These studies provide insight into the relation between bullying victimization and mattering but are limited to school-specific mattering. Feelings of significance within specific contexts (e.g., community, school, family) may not capture global mattering perceptions, although they may be related [33]. Moreover, this research has largely ignored variations across social identity groups, namely gender identity and sexual orientation.

### 1.2. Gender Identity, Sexual Orientation, Mattering, and Bullying Victimization

Bullying victimization may be particularly damaging to the sense of mattering of gender and sexually diverse youth. Gender and sexually diverse youth face additional, unique, and chronic forms of social and structural stressors and barriers associated with prevailing cis-heteronormativity and identity-based stigma, prejudice, and discrimination, as postulated by minority stress theory [48,49]. Bullying is no exception with decades of research illustrating that youth of varying gender identities [4,5,7] and sexual orientations [6,8] are bullied at alarming rates (44 to 77 percent) [8,50,51,52] compared to the global prevalence of bullying across youth (30 percent) [46]. This abuse can be characterized as being homophobic and transphobic in nature. Such high levels of bullying are thought to stem from peer attempts to maintain group norms that are informed by societal cis-heteronormative roles and values, see [53,54,55]. These inequities are likely to be associated with lower mattering among bullied gender and sexually diverse youth given a breadth of research that already demonstrates how bullying experiences threaten the self-perceptions and psychological adjustment of 2SLGBTQIA+ individuals, including its associations with lower self-esteem [56,57] and greater anxiety, depression [58,59,60], and substance use [61,62]. Moreover, general sociocultural messages and practices that enforce the devaluation and discrediting of minority groups, such as those of diverse gender identities and sexual orientations, likely further place these youth at risk for lower mattering [44]. As such, the combination of general and minority specific stressors may place bullied gender and sexually diverse youth at elevated risk for low mattering compared to their bullied non-2SLGBTQIA+ peers.

Youth who possess multiple diverse social identities may experience further differences in mattering because of bullying. Intersectionality provides a framework for understanding how the intersection of multiple social identities can jointly produce distinct life experiences within social hierarchies, including compounding disparities in marginalization and well-being [48,49,63,64]. Indeed, previous studies demonstrate such differences in bullying exposure [5,65] and associated psychological adjustment [5,60] among those of intersecting gender identity and sexual orientations. Although differences in mattering by gender identity [19,43,66,67,68] and sexual orientation [32,69] have been explored independently, it remains unclear whether the relation between bullying victimization and mattering differs across intersecting gender identity and sexual orientation groups. Further, researchers have exclusively relied on binary conceptualizations of gender, possibly due to a lack of statistical power, thereby overlooking how gender diversity may intersect with sexual orientation to shape youth’s experiences of mattering in relation to bullying victimization. Accordingly, we examined whether there were experiential differences in mattering among bullied youth of varying gender identities and sexual orientations.

### 1.3. Present Study

Our aim was to examine the association between bullying victimization and the sense of mattering of Canadian youth in Grades 7–12 and determine whether this association was moderated by the intersection of gender identity and sexual orientation using cross-sectional data. To expand upon previous context-specific mattering instruments used in the literature, participants were provided with a general measure of mattering to explore broader perceptions of significance to others.

We hypothesized a main effect of bullying victimization predicting mattering, such that youth who experience greater bullying victimization would report lower perceptions of mattering across gender identities and sexual orientations (H1). Based on minority stress frameworks [48,49], we further hypothesized that sexual orientation would moderate the association between bullying victimization and mattering, such that more frequently bullied sexually diverse youth would report lower mattering than bullied straight youth, controlling for gender identity (H2a). Similarly, we hypothesized that gender identity would moderate this association, such that more frequently bullied gender diverse youth would report lower mattering than bullied girls and boys, controlling for sexual orientation (H2b). Finally, consistent with an intersectional framework [63,64], we hypothesized a three-way interaction between bullying victimization, gender identity, and sexual orientation, such that this association would be strongest among youth with intersecting marginalized identities (i.e., greatest effect for sexually gender diverse youth, followed by sexually diverse girls, sexually diverse boys, straight gender diverse youth, straight girls, straight boys; H3).

## 2. Materials and Methods

### 2.1. Procedures and Participants

A subsection of Time 1 cross-sectional data were used from the Health and Peer Relations Study (HPRS), an ongoing longitudinal population-based survey that tracks Grade 4 to 12 students’ perceptions of school climate, school safety, physical and mental health, and peer relationships of select school boards in Ontario, Canada. For the current study, data were collected in the Spring of 2025. Participation required passive parental consent and active student assent/consent. Students were informed about the study’s purpose, right to voluntary participation, and assured of the anonymity of their responses. Students were provided the option to skip questions or withdraw from the study at any time. Those without parent consent or student assent/consent were asked to work independently until other students were finished the survey or were provided with a mock survey. Ethics approval for the HPRS was obtained from the University of Ottawa’s Research Ethics Board and the participating school boards.

**Figure 1:**
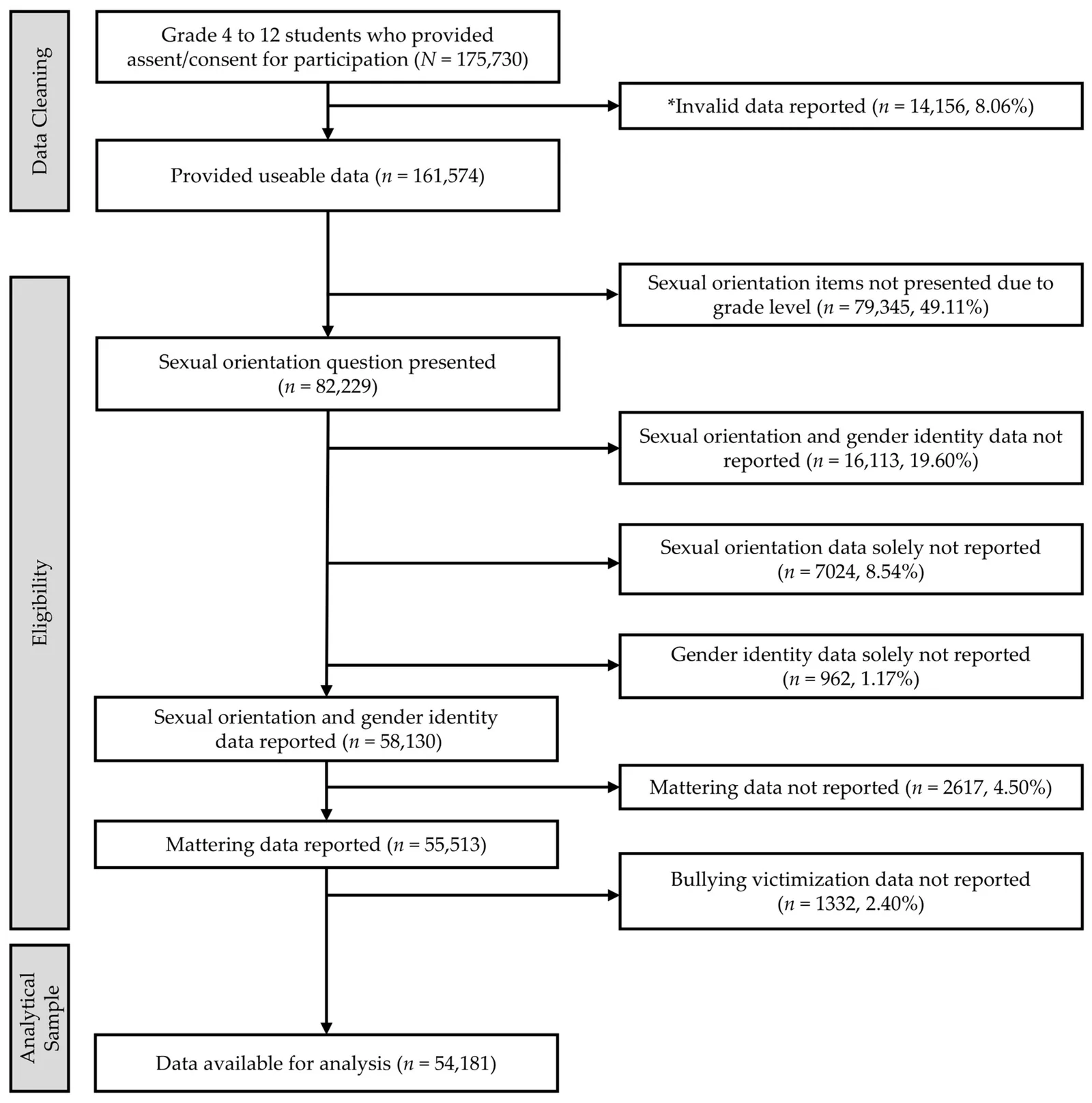
A flow chart regarding data cleaning, eligibility criteria, missing data, and the final analytical sample is presented. The percentage of missing data is also reported. * indicates that the invalid data category includes those who closed the survey after assent or skipped all survey questions (*n* = 5027) and those flagged as invalid for being dishonest, inattentive, or having an inconsistent grade (*n* = 9129).

Participants at Time 1 Spring consisted of 175,730 students in Grades 4 to 12 who provided assent/consent for the survey. Using multi-method response validity screening techniques recommended when conducting youth surveys [70,71,72], 5% (min = 3.5%, max = 7.6%) of students across school boards were removed because they reported being dishonest on the survey, answered three or more attention questions incorrectly, and/or had an invalid or mischievous open-ended response that was removed after agreement between a research associate and/or research assistant(s). From this, 161,574 individuals provided usable data. For the current study, the analytic sample was selected based on having scores on all variables of interest (i.e., sexual orientation, gender identity, mattering, and bullying victimization; *n* = 54,181). Only students in Grades 7–8 (in some school boards) and 9–12 were presented with gender diversity and sexual orientation items. Data cleaning, eligibility criteria, missing data, and final analytical sample can be found in Figure 1.

***General Mattering.*** Mattering was assessed using an adapted version of the five-item General Mattering Scale (GMS) [10,73,74]. This self-report measure asked participants to indicate the extent to which they believe they matter to others (e.g., “How important are you to others?”, “How interested are people generally in what you have to say?”) on a 4-point frequency scale, ranging from 1 (*not at all*), 2 (*a little*), 3 (*somewhat*), to 4 (*a lot*). The wording of an item from the original scale (i.e., “How much would you be missed if you went away?”) was modified for use in the present study (i.e., “How much do you feel others would miss you if you went somewhere else?”). An additional sixth item was added based on recent studies (i.e., “How much do other people show they care about you?”) [74]. As a sensitivity analysis, all analyses were conducted with the original 5-item scale and the newer 6-item scale. The results were the same, and as such, we report the results using the 6-item scale. Responses were averaged to create a total score to be consistent with previous studies and to allow for this score to be computed when up to two items were missing [67]. Internal consistency was good (*α*  =  0.88).

***Bullying Victimization.*** A five-item self-report scale adapted from the Olweus Bully/Victim Questionnaire [36,37,75] was used to measure students’ experiences of bullying. At the recommendation of previous studies [76], a definition of bullying (intentionality, repetition, and presence of a power imbalance by a single or group of aggressors) was provided to allow participants to distinguish experiences of bullying more accurately from those involving teasing and aggression. Participants were then asked about their general experiences with bullying victimization (i.e., “Since the start of the school year [September], how often have you been bullied by another student(s)?”). The remaining four questions asked students to indicate the extent to which they experienced different types of bullying, including general, physical, verbal, social, and cyber, on a 5-point frequency scale, ranging from 0 (*not at all*) to 4 (*many times a week*). Mean scores were created, allowing for up to one missing item. This scale has been widely used [36,37]. In the present study, this measure demonstrated good internal consistency (*α*  =  0.83).

***Demographic information.*** Using multiple-choice and checkbox response options to optimize inclusivity and sensitivity, participants were asked to report their gender identity, sexual orientation, racial/ethnic background, and current grade level. Demographic items and options were individually curated to align with specific school board priorities and requests and thus had slight item variations. These items were developed with guidance from school board LGBTQ+ advisory committees as well as the Ontario Anti-Racism Directorate’s data standards [77].

Where applicable, definitions of sexual orientation and gender identity were presented. Students were then asked to select the category(ies) that best described their sexual orientation and gender identity and shown a series of responses (e.g., gender identity: agender, boy, bigender, demiboy, demigirl, genderfluid, genderqueer, girl, non-binary, questioning, or do not know; e.g., sexual orientation: asexual, bisexual, gay or lesbian, pansexual, queer, questioning, straight, or heterosexual). Individuals were also provided an open-ended response option (i.e., “Other, in your own words:”) for those who preferred to describe their identity. Definitions of asexual, bisexual, gay or lesbian, pansexual, questioning, as well as agender, genderfluid, non-binary, and questioning were provided at the request of the participating school boards. All demographic items included a “skip” response option, allowing participants to choose not to answer any question for any reason. Open-ended responses were coded by two graduate-level research assistants. Terms identical to prelisted options were placed into their respective categories to ensure comparability. Within sexual orientation and gender identity, the “Other” category consisted of open-ended responses that expressed a non-exclusive heterosexual, girl, or boy identity but could not be placed into a prelisted category. All open-ended response coding was checked and agreed upon by two research assistants. For the current study, sexual orientation categories were dichotomized into straight or heterosexual and sexually diverse because of statistical power constraints. Gender identity categories were collapsed into girl, boy, and gender diverse due to similar limitations. Those who responded “Questioning” or could not be placed into a prelisted category were placed into the sexually diverse or gender diverse category, respectively. Those who selected “do not know” were indicated as missing given that we were unable to distinguish whether this referred to their conceptual understanding of the question of their sexuality or gender identity.

### 2.2. Statistical Analysis

The Statistical Package for Social Sciences (SPSS) version 29 was used to calculate descriptive statistics on gender identity, sexual orientation, grade level, and race/ethnicity, as well as mattering and bullying victimization.

Analyses were performed in Mplus (Version 8.11) [78]. Models were estimated using maximum likelihood estimation with robust standard errors (MLR), which provides more reliable estimates when data may not fully meet normality assumptions. Because students were nested within schools, analyses accounted for the possibility that students from the same school may have more similar responses than students from different schools. This was addressed using the TYPE=COMPLEX option in Mplus, which adjusts standard errors for this clustering.

Missing data on covariates were handled using full information maximum likelihood (FIML), which allows all available data to contribute to the analyses rather than excluding participants with incomplete information. Covariate means were estimated as part of this process.

A series of nested saturated regression models predicting mattering were estimated. Model 1 (M1) included centered covariates (grade and ethnicity) and the main effects of bullying victimization, sexual orientation, and gender identity. Model 2 (M2) added all two-way interactions among these variables. Model 3 (M3) added the three-way interaction between bullying victimization, sexual orientation, and gender identity. At each step, Wald chi-square tests were conducted to determine whether the newly added interaction terms significantly improved the model. This was done by constraining the newly added parameters to zero and testing whether these constraints significantly reduced model fit. Results from the final model (M3) are reported.

If significant interaction effects were identified, follow-up multi-group analyses would be conducted to examine whether the association between bullying victimization and mattering differed across groups defined by intersecting sexual orientation and gender identity categories. Specifically, these analyses compared the magnitude of the association between bullying victimization and mattering across identity groups.

To further interpret these findings, differences in average levels of mattering across identity groups were examined by comparing group-specific intercepts, in addition to differences in the association between bullying victimization and mattering.

## 3. Results

### 3.1. Descriptive Statistics

#### 3.1.1. Sample Characteristics

The characteristics of the analytic sample are displayed in Table 1. Most of the analytic sample identified as a girl (50.3%; gender diverse, 3.5%), racially/ethnically diverse (65.2%), straight (85.2%), and between Grades 9–12 (69.0%). Of the total analytic sample, 14.8% identified as sexually diverse. There were significant variations in sexual orientations (sexually diverse, straight) across gender identities (gender diverse, girl, boy), χ^2^(2, 54,181) = 10,738.11, *p* < 0.001, Cramer’s V = 0.45. A greater number of gender diverse youth (92.4%) identified as sexually diverse, compared to girls (17.1%) and boys (6.3%; proportion of girls also higher than boys).

#### 3.1.2. Missing Data

Percentages of missing data are shown in Figure 1. Analyses were conducted to examine if there were any key variable (i.e., sexual orientation, gender identity, bullying victimization, mattering) differences between those who were included in the analytic sample and those who were eligible but not included due to missingness on key variables. Chi-square tests of independence and independent samples *t*-tests were used for these analyses. A Cramer’s V value of >0.10 and a Cohen’s d value of >0.20 were used as cutoff values for statistically meaningful differences between the analytic sample and non-selected participants. Using these values, the Chi-square tests of independence indicated that the decision to provide data was not related to sexual orientation, *χ*^2^(1, 59,092) = 67.26, *p* < 0.001, Cramer’s V = 0.03, or gender identity, *χ*^2^(2, 65,172) = 15.99, *p* < 0.001, Cramer’s V = 0.02. Independent samples *t*-tests showed that the decision to provide data was also not associated with bullying victimization, *t*(28,287.83) = 6.27, *p* < 0.001, *d* = 0.06, or mattering, *t*(21,318.92) = −15.13, *p* < 0.001, *d* = −0.15. Thus, data were assumed to be missing at random.

### 3.2. Moderation Analyses

In M1, the covariates (grade and ethnicity) and main effects of bullying victimization, sexual orientation, and gender identity were each statistically significantly related to mattering, and the model accounted for 11.6% of the variance in mattering, *R*^2^ = 0.115, S.E. = 0.004, *p* < 0.001. Older and racially/ethnically diverse youth were more likely to report lower mattering than younger and white peers, respectively. Higher bullying victimization was associated with lower mattering. Compared to straight youth, sexually diverse youth were more likely to report lower mattering. Girls reported higher mattering than boys and gender diverse youth, and boys reported higher mattering than gender diverse youth. In M2, 2-way interactions were added and, as a set, were associated with mattering, Wald χ^2^(5) = 42.223, *R*^2^ = 0.116, S.E. = 0.004, *p* < 0.001. Specifically, the two-way interaction of gender and victimization was statistically significant, Wald χ^2^(2) = 39.580, *p* < 0.001, with the magnitude of victimization predicting mattering stronger for girls than gender diverse youth and boys. The interaction of sexual orientation and victimization was not statistically significant, Wald χ^2^(1) =1.132 *p* = 0.288. In M3, a 3-way interaction for victimization × sexual orientation × gender identity was tested by adding two additional parameters representing differences in the association between bullying victimization and mattering across intersecting identity groups. Together, these parameters tested whether the relationship between victimization and mattering varied as a function of both sexual orientation and gender identity. The set of parameters was associated with mattering, Wald χ^2^(2) = 6.331, *R*^2^ = 0.116, S.E. = 0.004, *p* < 0.001. Parameters from this final model are presented in Table 2.

#### Interaction Effects

**Figure 2:**
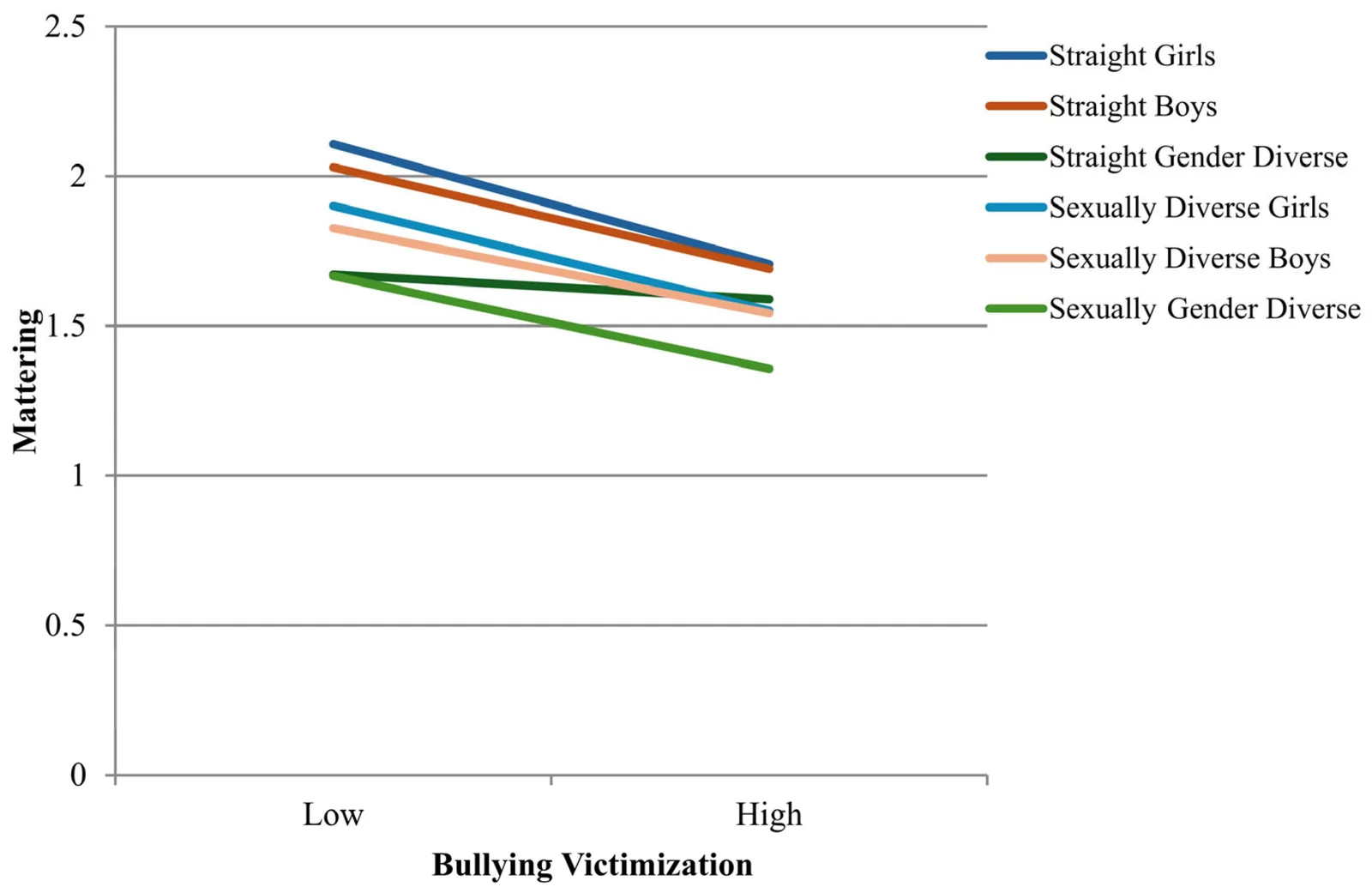
Gender identity (girl, boy, gender diverse) and sexual orientation (straight, sexually diverse) differences in bullying victimization and youth sense of mattering to others. High bullying victimization is plotted at one standard deviation above the mean. Low bullying victimization is plotted at the minimum score rather than one standard deviation below the mean because one standard deviation below the mean was lower than the scale minimum. Higher scores on mattering suggest greater perceptions of significance to others. High scores on bullying victimization suggest more frequent bullying victimization.

To interpret significant interaction effects, follow-up multi-group analyses were conducted. These analyses compared the strength of the association between bullying victimization and mattering across groups defined by intersecting sexual orientation and gender identity categories. Bullying victimization was statistically significantly associated with mattering for all groups, except for straight gender diverse youth (see Table 3 for parameter estimates). The magnitude of this association was strongest for straight girls, followed by sexually diverse girls, straight boys, and sexually gender diverse youth, as well as sexually diverse boys. The association between bullying and mattering was strongest for straight girls in comparison to all other groups. This association was stronger for straight boys than for straight gender diverse youth but did not differ from all other groups (i.e., sexually diverse girls, sexually diverse boys, or sexually gender diverse youth). Sexually diverse girls showed a stronger association than sexually diverse boys but did not differ from sexually gender diverse youth. Finally, the association was stronger for sexually gender diverse youth than for their straight gender diverse peers. These associations are displayed in Figure 2.

To better understand differences between groups, we also examined whether groups differed in their average levels of mattering (i.e., the intercepts of mattering) independent of bullying victimization. These group differences are presented in Table 3. Straight girls reported the highest levels of mattering, followed by straight boys, then sexually diverse girls, sexually diverse boys, and straight gender diverse youth, with sexually gender diverse youth reporting the lowest levels. Straight girls reported the highest level of mattering compared to all other groups. Straight boys reported greater mattering than straight gender diverse youth, sexually diverse girls, sexually diverse boys, and sexually gender diverse youth. Sexually diverse girls reported greater levels of mattering compared to sexually diverse boys and sexually gender diverse youth. Sexually diverse boys demonstrated greater mattering compared to their sexually gender diverse counterparts. However, straight gender diverse youth did not significantly differ from sexually diverse girls, sexually diverse boys, and sexually gender diverse youth.

### 3.3. Summary

Overall, youth’s sense of mattering depended on their exposure to bullying, sexual orientation, and gender identity, with greater bullying victimization being associated with lower sense of mattering across most sexual orientation and gender identity groups. Although this negative relation was most pronounced for straight girls, the association remained largely uniform across most other intersecting identity groups. Notably, straight girls and boys reported the highest average levels of mattering, whereas sexually and gender diverse youth reported consistently lower, relatively comparable levels overall.

## 4. Discussion

We explored variations in mattering as a function of bullying victimization at specific intersections of gender and sexual identity. Overall, findings provided partial support for our hypotheses and highlight a more complex pattern of identity-based differences than anticipated.

Consistent with H1, bullying victimization was associated with lower perceptions of mattering across most groups and aligns with prior work indicating that bullying undermines youth social significance perceptions [32,34] and self-conceptualizations [42]. Our findings underscore the importance of peer interactions and relationships during adolescence, with repeated peer maltreatment associated with weaker youth mattering perceptions. Socio-ecological diathesis models propose that youth with certain biological, cognitive, and social vulnerabilities who are exposed to bullying may have an increased tendency to internalize these experiences and thereby shape beliefs about themselves, others, and the world [79]. Given that the nature of peer interactions and relationships holds such high importance in their lives, bullying may strongly communicate and be particularly influential to how youth see their value, importance, and significance in the eyes of others more generally [33,45]. However, there was an absence of an association between bullying and mattering among straight gender diverse youth, indicating that the relation between bullying victimization and mattering is not uniform across intersecting identity groups.

Despite most youth with bullying experiences reporting lower mattering across gender identity and sexual orientation groups, there were some unexpected results within groups. Specifically, straight and sexually diverse youth did not differ in terms of the association between bullying and mattering perceptions, contrary to H2a and minority stress frameworks. When considering within-group differences within gender identity, greater bullying was more strongly related to lower mattering perceptions among girls compared to their boy and gender diverse counterparts, contrary to H2b. These findings suggest that social disadvantage based on sexual or gender identity does not consistently correspond with stronger observed associations between bullying victimization and mattering. However, because we found evidence of a three-way interaction, findings suggest that the association between bullying and mattering depended on youth sexual orientation and gender identity rather than independently.

Similar to the results of H2, although there was a three-way interaction between bullying victimization, gender identity, and sexual orientation, the association was not strongest for youth with multiple marginalized identities (i.e., sexually gender diverse youth followed by sexually diverse girls, sexually diverse boys, straight gender diverse youth, straight girls, straight boys) as hypothesized (H3). Rather, the strongest association between bullying victimization and mattering emerged among straight girls compared to all other groups. Bullying victimization on youth’s sense of mattering was largely similar for all other groups, with the exception being straight gender diverse youth, wherein bullying was not associated with mattering. This pattern differs from theoretical perspectives suggesting that multiple marginalized identities may correspond with compounded social disadvantages under particular sociocultural conditions [63,64].

However, the analyses of intercepts provided a more complete account of these unexpected and complex group and intersectional patterns. Straight girls reported the highest levels of mattering compared to all groups, followed by straight boys. Further, sexually diverse girls, sexually diverse boys, sexually gender diverse youth, and straight gender diverse youth tended to experience similar lower levels of mattering. These findings appear to suggest a social gradient, in which straight girls and boys tend to believe more strongly that they are important and valued by others, whereas those who are sexually and/or gender diverse reported similarly lower average levels of mattering. This gradient may help to contextualize the observed within-group and interaction effects by highlighting how social positioning and identity-based experiences may relate to differences in average mattering and responses to social stressors. From an intersectional perspective, youth occupying more socially advantaged positions (e.g., being a girl or boy as well as straight) within cis-heteronormative social systems may be more likely to experience greater alignment between their identities and dominant social values, which may correspond with stronger perceptions of social importance. As a result, bullying victimization may represent a stronger deviation from their typical expectations of social value and significance.

Conversely, youth with marginalized sexual and/or gender identities may navigate social contexts in which their identities are less consistently reflected or affirmed, which may be associated with lower average expectations of being socially valued. As mentioned, sexually and gender diverse youth are disproportionately exposed to multiple forms of interpersonal and structural stigma [48,49]. Outside of bullying victimization, sexually and gender diverse youth experience elevated exposure to trauma, with approximately 55% reporting at least one form of childhood adversity, commonly sexual and physical abuse, before the age of 18 [80]. Further, 2SLGBTQIA+ youth experience additional distal (external; e.g., prejudice, discrimination) and proximal (internal; e.g., internalized stigma, hypervigilance) minority stressors associated with stigmatization [48,49], including actual or threatened physical and/or sexual assault [81,82]. These forms of stigma are also experienced across multiple social contexts [83]. Although discrimination based on sexual and gender identity is prohibited under the Canadian Human Rights Act [84], more recent public discourse suggests that acceptance of sexually and gender diverse individuals in Canada has faced increasing challenges, attributed in part to the influence of anti-2SLGBTQIA+ discourse originating from the United States [85,86]. These broader interpersonal and structural contexts may contribute to differences in average perceptions of social value among sexually and gender diverse youth. Within this context, weaker and less differentiated associations between bullying victimization and mattering among sexually and/or gender diverse groups compared to more socially advantaged groups may reflect differences in average perceptions of social value and expectations of being valued by others, rather than indicating reduced susceptibility to the harmful effects of bullying. Instead, these patterns may suggest that the relation between bullying and mattering is embedded within broader social contexts.

Disparities in the association between bullying victimization and mattering emerged when considering intersecting identities, notably so for straight girls who reported the highest levels of mattering and whose lower mattering perceptions were more strongly tied to bullying victimization frequency compared to other groups. Such results align with previous research that find girls have a higher sense of mattering compared to boys [19,43,68] and may speak to the tendency for girls to be sensitive to and have a self-concept that is interpersonally focused [87,88,89], as suggested by previous research [68]. Accordingly, bullying may threaten girls’ social expectations, values, and self-conceptualizations, and thus be more strongly related to their perceived social importance.

The lack of association between bullying and mattering for straight gender diverse youth in contrast to all other groups in our sample was unexpected. This lack of an association may suggest that gender diverse youth who identify as straight may face unique stressors and barriers associated with lower mattering outside of bullying victimization. However, of the 3.5% of our sample that identified as gender diverse, approximately 8% identified as straight, which is in stark comparison to approximately 92% of their counterparts who identified as sexually diverse. Given this comparatively small sample size, our analysis of straight gender diverse youth may have lacked the statistical power needed to detect the true strength of an effect.

Thus, these patterns may be understood as reflecting how broader systems of social privilege and marginalization intersect with experiences of bullying to shape perceptions of social value. Rather than indicating that bullying is less harmful for sexually and/or gender diverse youth, the findings may suggest that the relation between bullying victimization and mattering could be situated within broader social contexts that influence how youth perceive and interpret their social worth. As such, it may be that in the context of bullying victimization, possessing multiple marginalized identities may not multiply an individual’s risk for lower mattering in a straightforward manner as minority stress and intersectionality frameworks may suggest. However, because we did not directly measure structural and contextual intersectional factors, explanations related to youth social positioning and identity-based experiences can only be theorized.

Alternative explanations for these unexpected findings should also be considered, including methodological factors. For example, our unanticipated within-group and intersectional differences may also stem from methodological considerations. For example, we used a general rather than bias-based bullying measure, which is bullying that is motivated by an individual’s perceived or actual affiliation with a stigmatized group (e.g., race, ethnicity, gender, sexual orientation, disability status) [90]. Bias-based bullying is a relevant concern for youth of diverse social identities, including for gender and/or sexually diverse youth [5]. Further, bias-based bullying is associated with worse outcomes including school avoidance [91], unwellness [92], and mental health and substance use challenges [93]. Given that our measure did not distinguish between general and bias-based bullying experiences, our results may underestimate the extent to which bullying specifically related to sexual orientation and/or gender identity contributes to differences in mattering across intersectional groups.

In sum, bullying victimization is associated with lower mattering for most youth. However, this relation varies in complex ways across intersecting identities once average levels in mattering are considered. Overall, findings suggest a dual pattern in which differences in average levels of mattering may contextualize variability in the observed association between bullying victimization and mattering. Specifically, bullying victimization was more strongly associated with lower mattering among some groups reporting higher average levels of mattering.

### 4.1. Strengths and Limitations

Several limitations need to be stated when considering the results of our study. First, the cross-sectional and correlational nature of our study does not allow us to claim a causal relation between bullying victimization and a sense of mattering. Rather, there may be a bidirectional relation between these constructs, such that lower mattering may be a risk factor for future bullying victimization and vice versa.

Second, all measures in the present study were self-reported. Although mattering is inherently a subjective experience [10,12,17], self-reported bullying victimization may differ from behaviour-based or peer-reported assessments. Self-reported experiences of victimization are meaningful indicators of youth well-being [94,95] and demonstrate convergence with other bullying measures [95]. However, different measurement approaches may capture distinct aspects of bullying and yield different estimates of victimization. Consequently, the exclusive reliance on self-report measures may have influenced the observed associations.

Third, the relatively small number of straight gender diverse youth, combined with substantial variability in bullying victimization and mattering scores, may have reduced the statistical precision and stability of the intersectional estimates for this subgroup.

Fourth, sexual orientation and gender identity were collapsed into binary and ternary variables due to statistical power constraints. Although necessary, this approach may have obscured important heterogeneity in experiences across specific identities. For example, youth questioning their sexual orientation and/or gender identity were grouped with sexually and gender diverse participants rather than examined as a distinct subgroup. Emerging evidence suggests that questioning youth may experience unique patterns of victimization and psychosocial outcomes that differ from straight, cisgender and LGB+ and TGD+ youth, e.g., [96,97,98]. Consequently, collapsing sexual orientation and gender identity categories may have masked important subgroup differences and obscured whether specific identities may have disproportionately contributed to the observed association between bullying victimization and mattering in our study.

Fifth, we were unable to examine additional intersecting social identities, including race/ethnicity and disability status, due to statistical power constraints. This is noteworthy given that intersecting systems of marginalization may shape youths’ experiences of bullying. For example, sexually and gender diverse youth of colour may face unique forms of victimization arising from the intersection of racism, sexism, and homo/bi/transphobia [97,99] and are disproportionately targeted based on race, weight/appearance, and disability [100], a pattern that may also extend to gender diverse youth. As such, the present findings may underestimate heterogeneity in bullying experiences among youth of multiple diverse social identities.

Finally, our findings should also be interpreted considering several unmeasured contextual factors. Structural inequalities, school policies, family acceptance, social stigma, and community characteristics were not directly assessed and therefore cannot explain the observed associations in this study. Rather, these factors represent plausible mechanisms suggested by intersectionality theory and prior research that warrant direct examination in future studies.

Despite these limitations, this study adds to the literature in major ways. Our results stem from a large, population-based sample and thus can be generalized to the broader Canadian population. This is noteworthy given that it is difficult to collect representative data pertaining to minority populations, such as gender and sexually diverse youth. Further, the use of multi-method data screeners for detecting and removing invalid and mischievous responses in our sample adds to the integrity of our findings, especially since such responders often falsely claim 2SLGBTQIA+ membership and consequently inflate reports of experiential disparities in these communities [70]. Our study also used a measure of global mattering, expanding upon previous studies. Lastly, to our knowledge, our study is the first to explore gender identity and sexual orientation intersectional differences in the association between bullying victimization and sense of mattering among youth, giving further testament to the contribution of this study.

### 4.2. Implications

The lower levels of mattering associated with bullying victimization and those observed among gender and sexually diverse youth have wider implications. Specifically, weak perceptions of mattering have been associated with academic factors, such as increased academic exhaustion [18], risk of dropping out of school [101], lower school connectedness [102], and lower perceptions of school climate [67], as well as mental health consequences, including loneliness [18,103], anxiety, depression, and social media addiction [102]. Even more concerning is that both bullying victimization [104] and low levels of mattering [105] independently predict increased risk of suicidality among youth. Suicidality is also elevated among 2SLGBTQIA+ youth, with experiences of bullying being associated with increased risk in this population [106]. Conversely, elevated perceptions of significance to others can buffer against mental health outcomes and suicidality stemming from peer victimization [107] and among 2SLGBTQIA+ youth [106]. Thus, youth who experience bullying victimization and/or occupy marginalized social positions may have less consistent access to the benefits of this highly protective resource and may consequently be at heightened risk for academic, psychological, and life-threatening consequences.

## 5. Conclusions

Most youth who were targets of bullying tended to have weaker perceptions that they mattered to others, regardless of gender identity and sexual orientation. However, the strength of this association varied in important ways across intersecting identities: bullying victimization was most strongly related to lower mattering among straight girls, while the strength of these associations was largely similar across other groups and absent among straight gender diverse youth. At the same time, average differences revealed a clear social gradient, with sexually and gender diverse youth reporting the lowest levels of mattering overall. These findings suggest that although bullying is broadly harmful, disparities in mattering may reflect underlying structural inequalities rather than differences in sensitivity to victimization, whereby youth with greater initial social advantage may show stronger associations between bullying victimization and mattering. As suggested by Flett and colleagues [17,108], focusing on incorporating messages and actions that bullied and gender and/or sexually marginalized youth are valued, significant, cared about, and depended on at the policy, community, school, classroom, family, and individual level is an important consideration for supporting these youth, especially for those who need to know that they matter the most. Future research should continue to explore bullying and mattering among youth, with a focus on intersectional differences. Investigating structural and contextual dimensions of intersectionality, such as socioeconomic status, family support, school climate, and regional differences, would improve our understanding of the complex social and relational factors that shape the mattering experiences of bullied and non-bullied youth of intersecting identities. Examination into the experiences of straight gender diverse youth would build upon the current literature.

## Figures and Tables

**Table 1:** Descriptive statistics and sociodemographic characteristics of participants.

Characteristics	Total (*N* = 54,181)	Girl (*n* = 27,235)	Boy (*n* = 25,030)	Gender Diverse (*n* = 1916)	Straight (*n* = 46,183)	Sexually Diverse (*n* = 7998)
Bullying Victimization	0.45 (0.65)	0.43 (0.61)	0.45 (0.69)	0.69 (0.87)	0.43 (0.63)	0.55 (0.73)
Mattering	1.88 (0.70)	1.91 (0.69)	1.88 (0.69)	1.55 (0.74)	1.92 (0.69)	1.70 (0.72)
Gender Identity						
Girl	50.3%				48.9%	58.2%
Boy	46.2%				50.8%	19.6%
Diverse	3.5%				0.30%	22.1%
Sexual Orientation						
Straight	85.2%	82.9%	93.7%	7.6%		
Diverse	14.8%	17.1%	6.3%	92.4%		
Race/ethnicity						
White	34.8%	33.7%	35.2%	45.6%	33.8%	40.5%
BIPOC	65.2%	66.3%	64.8%	54.4%	66.2%	59.5%
Grade Level						
7–8	31.0%	30.0%	32.3%	28.4%	31.9%	26.0%
9–12	69.0%	70.0%	67.7%	71.6%	68.1%	74.0%

Means and standard deviations are provided for continuous variables (i.e., bullying victimization and mattering). Proportions for categorical variables are presented as percentages within each column (i.e., total, gender identity, sexual orientation, race/ethnicity, grade level). BIPOC = Black, Indigenous, People of Colour.

**Table 2:** Three-way interaction between bullying, gender identity, and sexual orientation with youth sense of mattering.

Source	*b*	*se*	*p*	*β*	*se*	*p*
Intercept	1.612	0.024	0.000	2.316	0.037	0.000
Race/Eth	–0.104	0.008	0.000	–0.071	0.006	0.000
Grade Level	–0.053	0.003	0.000	–0.120	0.006	0.000
Vic	–0.306	0.021	0.000	–0.287	0.020	0.000
Girl	0.173	0.021	0.000	0.124	0.015	0.000
Boy	0.142	0.027	0.000	0.102	0.019	0.000
SO	0.057	0.065	0.386	0.029	0.033	0.386
Vic*Girl	–0.031	0.023	0.180	–0.019	0.014	0.180
Vic*Boy	0.033	0.031	0.294	0.021	0.020	0.295
Vic*SO	0.220	0.101	0.029	0.185	0.085	0.029
Girl*SO	0.092	0.066	0.164	0.065	0.047	0.164
Boy*SO	0.074	0.067	0.268	0.053	0.047	0.268
Vic*Girl*SO	–0.247	0.098	0.012	–0.139	0.055	0.012
Vic*Boy*SO	–0.248	0.103	0.016	–0.155	0.064	0.016

Race/Eth = race/ethnicity (coded −0.652 = racial/ethnic diverse, centered; 0.348 = white, centered). Vic = bullying victimization (centered). Girl and Boy represent dummy codes for gender (gender diverse reference group). SO = Sexual orientation (coded 0 = sexually diverse; 1 = straight). * = interaction between variables. *b* = unstandardized beta. *se* = standard error. *β* = standardized beta.

**Table 3:** Group comparison of mattering intercepts and the association between bullying victimization and mattering.

Groups	Intercept Mattering	*se*	*p*	*b* Bullying Victimization → Mattering	*se*	*p*
Straight girls	1.943 ^a^	0.006	0.000	–0.365 ^a^	0.009	0.000
Straight boys	1.891 ^b^	0.006	0.000	–0.309 ^bc^	0.008	0.000
Straight gender diverse	1.637 ^cde^	0.064	0.000	–0.075 ^ef^	0.094	0.425
Sexually diverse girls	1.758 ^ce^	0.014	0.000	–0.318 ^b^	0.016	0.000
Sexually diverse boys	1.710 ^d^	0.019	0.000	–0.259 ^cdef^	0.025	0.000
Sexually gender diverse	1.540 ^e^	0.020	0.000	–0.282 ^bcde^	0.021	0.000

Different letter superscripts within columns (i.e., within mattering intercept; within association between bullying and mattering) represent statistically significant differences between groups (*p* < 0.05). Same letter superscripts within columns (i.e., within mattering intercept; within association between bullying and mattering) indicate non-significant differences between groups (*ps* > 0.05). For example, in the association between bullying and mattering, sexually diverse girls are statistically significantly different from straight girls but are not statistically significantly different from straight boys and sexually gender diverse youth. *se* = standard error, *b* = unstandardized beta.

## Data Availability

The raw data supporting the conclusions of this article will be made available by the authors upon reasonable request due to ethical restrictions.

## Abbreviations

The following abbreviations are used in this manuscript:

2SLGBTQIA+	Two-Spirit, lesbian, gay, bisexual, transgender, questioning, intersex, asexual, plus
H1	Hypothesis 1
H2	Hypothesis 2
H2a	Hypothesis 2a
H2b	Hypothesis 2b
H3	Hypothesis 3
HPRS	Health and Peer Relations Study
LGBTQ+	Lesbian, gay, bisexual, transgender, questioning, plus
SPSS	Statistical Package for Social Sciences
BIPOC	Black, Indigenous, People of Colour
Race/Eth	Race and ethnicity
Vic	Bullying victimization
SO	Sexual orientation
Vic*Girl	Bullying victimization x girl interaction
Vic*Boy	Bullying victimization x boy interaction
Vic*SO	Bullying victimization x sexual orientation interaction
Girl*SO	Girl x sexual orientation interaction
Boy*SO	Boy x sexual orientation interaction
Vic*Girl*SO	Bullying victimization x girl x sexual orientation interaction
Vic*Boy*SO	Bullying victimization x boy x sexual orientation interaction
LGB+	Lesbian, gay, bisexual, plus
TGD+	Transgender, gender diverse, plus
